# Youth substance use service provider’s perspectives on use and service access in ontario: time to reframe the discourse

**DOI:** 10.1186/s13011-022-00435-9

**Published:** 2022-02-05

**Authors:** Farihah Ali, Cayley Russell, Frishta Nafeh, Claudia Chaufan, Sameer Imtiaz, Jürgen Rehm, Adrienne Spafford, Tara Elton-Marshall

**Affiliations:** 1 Centre for Addiction and Mental Health (CAMH), Institute for Mental Health Policy Research, #2035-33 Russell St, Toronto, M5S 2S1 Canada; 2 School of Health Policy and Management, York University, Toronto, M38 1P3 Canada; 3 Dalla Lana School of Public Health, University of Toronto, 155 College St, Toronto, ON M5T 3M7 Canada; 4 Centre for Addiction and Mental Health (CAMH), Campbell Family Mental Health Research Institute, 250 College St, Toronto, ON M5T 1R8 Canada; 5 Department of Psychiatry, University of Toronto, 1 King’s College Circle, Toronto, ON M5S 1A8 Canada; 6 Institute of Medical Science (IMS), University of Toronto, 1 King’s College Circle, Toronto, ON M5S 1A8 Canada; 7 Institut Für Klinische Psychologie Und Psychotherapie, Technische Universität Dresden, Chemnitzer Str. 46, 01187 Dresden, Germany; 8 Department of International Health Projects, Institute for Leadership and Health Management, I.M. Sechenov First Moscow State Medical University, Bol’shaya Pirogovskaya Ulitsa, 19c1, 119146 Moscow, Russia; 9 Center for Interdisciplinary Addiction Research (ZIS), Department of Psychiatry and Psychotherapy, University Medical Center Hamburg-Eppendorf (UKE), Martinistraße 52, 20246 Hamburg, Germany; 10 Addictions and Mental Health Ontario, 180 Dundas Street West, Toronto, ON M5G 1Z8 Canada; 11 Department of Health Sciences, Lakehead University, 955 Oliver Road, Thunder Bay, ON P7B 5E1 Canada; 12 Department of Epidemiology and Biostatistics, Schulich School of Medicine and Dentistry, Western University, Kresge Building, London, ON N6A 5C1 Canada; 13 Faculty of Medicine, School of Epidemiology and Public Health, University of Ottawa, Ottawa, ON K1G 5Z3 Canada

**Keywords:** Addiction, Adolescents, Alcohol, Cannabis, Harm Reduction, Public Health, Prevention, Substance Use, Stigma, Treatment

## Abstract

**Background:**

Age is a critical factor in substance use and related outcomes, with adolescence being a particularly sensitive period. Early initiation of substance use has been linked with higher risk for developing substance use disorders. In Ontario, Canada, substance use is common among youth, yet treatment is underutilized, suggesting the potential for an unmet need in terms of substance use care. Despite these challenges, there is limited research examining factors that contribute to youth substance use and youth-specific barriers to substance use care. To fill this knowledge gap, this study sought to include the unique perspectives of service providers who work directly with youth to examine these issues.

**Methods:**

We used a cross-sectional mixed-methods design to examine factors that contribute to substance use among youth and identify youth-specific barriers to substance use among a sample of 54 Ontario-based youth service providers. Data collection included an online survey completed by all study participants followed by qualitative interviews of a subsample of 16 participants. Data analysis included basic frequency tabulations for survey results and thematic qualitative analyses to identify common themes.

**Results:**

Licit substances were identified as the most commonly used among youth, where 94% of respondents identified cannabis use and 81% identified alcohol use. Thematic analyses identified the role of dominant substance use discourses in normalizing certain substances (i.e., cannabis and alcohol) while also endorsing stigmatizing beliefs and sentiments. According to youth service providers, the intersection of these two discourses simultaneously lead to an increase in substance use while deterring youth from seeking substance use care.

**Conclusions:**

Normalization and stigmatization are two dominant discourses around youth substance use, with important implications for public health interventions. Key public health strategies, as identified by participants, to reduce the overall negative effect of these factors include the need to reframe substance use discourse, from a moral failing to a public health issue and to educate youth about the impacts of use. To accomplish this goal educational campaigns to raise awareness around the health effects of use and address stigmatization are needed. Educational reforms are also needed to ensure that these programs are integrated into the school system.

## Introduction

Ontario, Canada, is home to approximately 2.97 million youth and young adults between the ages of 15–29, who make up an extremely diverse population [1]. Substance use among youth and young adults is very common in Ontario, and is markedly higher compared to older populations. For instance, 45% of Ontarians aged 18–29 reported past-year cannabis use in 2019, compared to 15% among those aged > 50 [2]. Additional data highlight the prevalent nature of substance use among youth in Ontario. For example, according to the 2019 Ontario Student Drug Use and Mental Health Survey (OSDUHS), over one-fifth (20.3%) of Ontario high school students reported any past-year drug use in 2019, with tobacco, alcohol and cannabis among the top substances most frequently used; and one-in-ten (11%) reported non-medical prescription opioid use [3]. Approximately 15% reported problematic substance use and less than one percent (0.7%) reported attending a substance use treatment program in the past year [3], indicating the potential for an unmet need in terms of substance use service and treatment access and uptake.

Adolescence, typically defined as the unique stage of human development between childhood and adulthood, brings about many social, mental and physical changes, including brain development, puberty, coping with new relationships, and evolving independence [4]. Adolescence and young adulthood are key life stages when behaviours and habits become established. Evidence shows that the earlier in life one initiates substance use, the higher the risk for developing substance use disorders, including dependence and addiction; this risk is further elevated when youth engage in frequent and/or polysubstance use [5, 6]. Adolescents are therefore vulnerable to negative effects of substance use, and are at an increased risk of developing long-term consequences as a result of use, such as mental health and/or substance use disorders [6, 7]. These issues underscore the need for youth to be able to access key prevention and treatment services and interventions for substance use in order to mitigate adverse outcomes from use.

There is no single cause of problematic substance use among youth, as it typically involves a variety of different factors (e.g., family, peer and societal influence, experiences of abuse or trauma, etc.) [8, 9]. However, youth who do develop problematic substance use habits and related issues often experience inadequate access to primary mental health and substance use care due to multiple access barriers and scarcity of evidence-based services tailored for youth specifically [10–12]. In Ontario, a variety of youth-based services that address substance use exist, and youth receive care in multiple settings; however, the youth substance use service system overall has been characterized as fragmented, untimely, under-resourced and not user-friendly [11–13]. Moreover, youth substance use services have been traditionally modeled after adult services, or are offered to youth within adult settings, which fail to provide developmentally-informed and effective services that are tailored to youth’s unique needs and may not always follow a harm reduction approach [14, 15]. While access to substance use services among youth is influenced by a multitude of macro and micro level factors [11, 12], previous literature has highlighted key barriers including individual-level barriers such as a lack of awareness and stigmatization, structural and administrative issues such as wait lists and burdensome paperwork, and physical barriers such as lack of transportation [12, 16, 17].

Importantly, the literature examining barriers to substance use service access among youth in Ontario has highlighted the critical role that substance use service providers play in the delivery of care [14, 15]. According to implementation sciences, service providers are important stakeholders in the delivery and uptake of evidence based interventions [18] and as such studies are increasingly relying on service provider perspectives to understand barriers and facilitators to care. Service providers have experience working directly with youth and have an understanding of the population’s needs, potential gaps in treatment and provision of care, challenges that surround treatment support, and barriers youth may face when attempting to access services [19]. Therefore, based on their knowledge and experiences they are experientially suited to understand these dynamics, and play a large role in influencing the delivery of appropriate policies and programs for youth. This places them in a unique position to be able to help identify and address problems, including potential access issues. Yet service providers are frequently left out of the conversation when it comes to understanding barriers to access and the needs of treatment-seeking youth. As such, there is a distinct need to include direct service providers’ perspectives when examining these questions.

Since preventing problematic substance use among youth is important for health and social outcomes, it is helpful, from the perspectives of service providers, to better understand the factors that contribute to youth substance use, as well as potential barriers that may deter youth from seeking support for substance use issues. To this end, this study sought to examine youth substance use from the perspective of direct service provider’s, to help identify potential solutions to better support youth who face substance use issues in Ontario.

## Methods

### Study design

The study utilized a cross-sectional mixed-methods data collection approach whereby publically-funded Ontario-based service providers (‘key informants’) participated in a confidential self-administered online survey via a survey management database tool called Research Electronic Data Capture (REDCap) [20]; and a sub-sample of interested key informants additionally partook in a semi-structured follow-up interview.

### Study instruments

The online survey was developed in consultation with a provincial network of researchers, service providers, policy makers, and the primary research team, which included an advisory council comprised of people with lived experience (PWLE). Questions focused on garnering key informant’s perspectives regarding commonly used substances among the youth they serve, challenges and barriers youth face when accessing substance use services, and youth’s needs in relation to interventions, treatment and service provision. Follow-up interview questions for the sub-sample of key informants were subsequently developed after analyses of the survey data in consultation with the research team and advisory council in order to further probe and contextualize key results that emerged from the survey.

### Recruitment

A member of the research team identified all youth-specific substance use service providers using the ConnexOntario public online database of over 5,000 government-funded addiction and mental health services in Ontario. There was a total of 152 organizations listed in the database, 46 of which had the same contact because they were from the same hub organization within the same geographic area. While the program names were different, the identified contacts were responsible for overseeing the programs, and were thus considered duplicates in the system. As such, they were excluded from the final list, resulting in 106 unique organizations/key informants to be contacted. An attempt to contact each organization was made via phone or email, and a key informant from 69 organizations expressed interest in participating in the online survey. The key informants subsequently provided their email address in order to receive the survey link. While 69 key informants expressed interest, a total of 54 key informants then completed the survey, representing a 51% response rate; key characteristics of organizations that did not respond or opted out were not assessed. At the end of the survey, participants had the opportunity to indicate if they were interested in participating in a follow-up interview, where they would then be re-contacted with further details regarding the interviews.

### Inclusion and exclusion criteria

Direct service providers employed within organizations that offered substance use treatment, support, or interventions to youth in Ontario who were fluent in either English or French language were invited to participate in the study. Direct service providers were defined as those who were responsible for service provision within their organization (e.g., program directors and managers, addiction counselors, etc.). Service providers who did not provide direct services to youth were excluded from participating, as were those who indicated that their service only provided mental health or problem gambling-related support.

### Data collection

Online surveys were administered between October 1^st^ and December 31^st^, 2019, and took approximately 20–30 min to complete. Follow-up key informant interviews occurred between February 7^th^ and 28^th^, 2020, and took between 30 min to one hour in length. A trained interviewer conducted the follow-up interviews either over the telephone or face-to-face for those who preferred and were located within the Greater Toronto Area. All interviews were confidential and audio-recorded. Key informants who participated in the follow-up interview received a $30 gift card as honoraria for their time and involvement in the study.

### Data analysis

Online survey data was analyzed using Statistical Analysis System (SAS Version 9.4 for Windows). Frequency and cross-tabulations were conducted to explore the responses, and graphs were created for data visualization. Audio-recorded qualitative interview data were transcribed verbatim and uploaded into qualitative data management software (NVivo version 12). Interview transcripts underwent an inductive thematic analysis whereby key themes based on our research questions were identified and subsequently coded into categories. Themes were included in final analyses when they were introduced or discussed by multiple participants.

### Ethics approval

The study was approved by the Centre for Addiction and Mental Health Research Ethics Board (REB # 063–2019) and York University (Certificate # STU 2020–010).

## Results

### Study sample characteristics

Table 1 outlines participant’s characteristics. A total of *n* = 54 Ontario-based youth service providers completed the online survey, and *n* = 16 participated in the follow-up interview. Based on Ontario’s interim transitional Local Health Integration Network (LHIN) regions (Watts et al., 2019), 22% of survey respondents were located in Central Ontario and 20% were in Northern Ontario. In terms of respondents, most participants were addiction counsellors (43%), followed by program directors (19%), and program managers (11%). With respect to service capacity, 57% of respondents identified that they served more than 50 youth on any given month. The average age of eligibility of services was 12, while the average cut-off was 24 years of age. The majority (94%) of participants identified cannabis as the most commonly used substance among youth they serve, followed by alcohol (81%).

**Table 1:** Study participants’ characteristics (*N* = 54)

Characteristics	% (*n*)
**Geographic Location**^**a**^	
Central Ontario	22% (12)
Northern Ontario	20% (11)
Other	57% (31)
**Service Provider Role**	
Addiction counsellor	43% (23)
Program Director	19% (10)
Program Manager	11% (6)
Intake Coordinator/Specialist	7% (4)
Program Coordinator	6% (3)
Clinical Supervisor	4% (2)
Addictions Physicians/Clinicians	4% (2)
Clinical Coordinator/Specialist	2% (1)
Outreach Specialist	2% (1)
Clinical Social Worker	2% (1)
Youth Addictions Worker	2% (1)
**Service Capacity**^**b**^	
≥ 51	57% (31)
≤ 50	43% (23)
**Most Commonly Used Substances**	
Cannabis	94% (51)
Alcohol	81% (44)
Cocaine	31% (17)
Tobacco	30% (16)
Opioids	19% (10)
**Service Enrolment Eligibility Age**	*Mean* (12 years)
**Oldest Age Eligible for Service**	*Mean* (24 years)

Note: Categories are not mutually exclusive

^a^Based on the Local Health Integration Networks (LHINs) classification system

^b^Number of youth that access the service in a given month

### Thematic results

Results from the surveys and interviews synergistically highlighted the impact of public discourse on substance use behaviours and treatment seeking patterns among youth. Key informants detailed their perspectives on how public discourse regarding substance use promotes the normalization of use (particularly for the most commonly used licit substances of cannabis and alcohol), while also endorsing stigmatizing beliefs and sentiments around substance use. Notably, and paradoxically, key informants suggested that the combination of these two outcomes (i.e., normalization and stigmatization) leads to an overall undesirable outcome, in that both discourses simultaneously result in an increase in substance use, while deterring youth from seeking treatment and support for their use. Key informants believed it is important to change the negative discourse around substance use while also educating those who use substances about the health effects of use. Education and awareness around the ways in which stigmatization can potentially perpetuate adverse health outcomes related to use among youth is also necessary. Below, we discuss each of these topics in-depth, using key informant quotations to illustrate essential points.

### Normalization of cannabis and alcohol use among youth

As cannabis and alcohol were reported as the most frequently used substances among youth in the survey, key informants were asked to elaborate on why they perceived this to be the case during the follow-up interviews. The main theme elicited related to how key informants perceived that public discourse essentially encourages cannabis and alcohol use through the normalization of these substances, which occurs via a number of mechanisms including the designation of these substances as licit, a lack of knowledge and education on the consequences of use, and social acceptability.

### Designation of cannabis and alcohol as licit substances

Key informants described how they perceived that the status of cannabis and alcohol as legal substances normalized their use and thus influenced youth’s decisions to use. Specifically, key informants perceived that the legal designation promotes use through logical reasoning that if the substance is legal, it must therefore be safe:

“I just think that because it’s legal, they think that there’s nothing wrong with it, because why would it be legal if it was bad for you?” (Key Informant 14)

Key informants explained how they believed that youth’s perceptions of cannabis and alcohol as legal and therefore non-problematic contributes to an increase in use, especially since youth consider the use of these substances a ‘safer’ choice compared to other illicit substances:

“The perceptions that youth maintain towards cannabis is that it’s low risk and relatively safe, so they have those perceived notions, so I think that increases the potential for experimentation.” (Key Informant 06).

Key informants described instances where due to the legalized status, use of cannabis and alcohol was not considered ‘drug use’ in the true sense, and indicated that many youth would not initially report their use of these substances until probed:

*“Sometimes I’ll have conversations regarding other substances and then it’s like ‘oh right, I smoke cannabis, do you consider that a drug?’ So again, youth are not considering cannabis a drug because of the way they were raised with it and not understanding it’s still a drug.” (Key Informant 09).*

Key informants also expressed that cannabis and alcohol are commonly used in social settings, which they perceived as a contributor to increased use. For instance, one informant discussed how they believed that legalization of alcohol contributes to increased use within social settings:

“Alcohol is definitely a problematic substance. The majority of clients here do have an alcohol dependency, and that was the reason to come into treatment. Again, because of the legalization of alcohol is a huge problem, especially with youth because it’s such a social thing to do, to go to parties, nightclubs and things like that. They become so dependent on the alcohol.” (Key Informant 04).

### Lack of knowledge and education

In addition to the role legalization plays in normalizing cannabis and alcohol use, key informants explained that they perceived that cannabis and alcohol use is normalized through a lack of education regarding potential adverse health and social impacts of these substances. Key informants suggested that many youth may not recognize or understand the potential long-term physical and psychosocial effects of using these substances, as well as the potential to lead to dependence. In terms of cannabis use specifically, participants expressed that they believed youth do not understand or consider its’ potential negative effects, including the possibility that it may lead to more frequent use, or use of other, possibly more problematic substances:

“Cannabis is absolutely problematic, because I think they’re minimizing the risk of it, because many of them think that it’s a natural substance. And secondly, they don’t think you can die from it, so it’s not seen as ‘harsh’. And I think the problem is, many people who I end up supporting longer term down the road, I find it’s a gateway drug to other substances, that’s what I’m finding.” (Key Informant 07).

Further, respondents described how they perceived that a lack of knowledge among youth leads to misconceptions about cannabis and alcohol being ‘safe’ and ‘low-risk’. In particular, based on their experiences and conversations with youth, participants described how youth are not taught about the association between adverse mental health outcomes (e.g., psychotic episodes) and cannabis use, and therefore do not grasp the potential severity of use:

“They don’t know how severe it is, especially with mental health. With cannabis, we have seen a lot of clients here that have come with mental health conditions, and all they do is over-consume marijuana, and then they go into a psychosis state of mind, which is very dangerous.” (Key Informant 04).

### Social acceptability

Key informants perceived that the normalization of cannabis and alcohol is perpetuated by the social acceptability of these substances. In particular, they mentioned how easily accessible and available cannabis and alcohol are, and how their accessibility and availability contributes to the normalization of these substances as socially acceptable:

“I believe it’s because of availability, because of it being normalized in our society as something that’s acceptable.” (Key Informant 05)

This theme was particularly salient for alcohol use, where key informants described how even though alcohol is illegal to purchase for most youth, it is still very accessible, leading to increased normalization of use:

*“I do feel [alcohol] is so easy to access. I mean yes, in Ontario the law says 19 years old, but it’s easy to have an older friend, or sibling, or ask somebody off the street to get it, or your parents have a cupboard full of it. I think it’s accessible.” (Key Informant 09)*

Key informants also described how alcohol is commonly used by household family members, at social gatherings, and how many parents do not recognize their own alcohol consumption patterns or behaviors – including storing alcohol in the house – as potentially problematic, in terms of increasing the likelihood of their children experimenting or viewing alcohol use as normalized behavior:

“That social component. I think because they see it in the house, right? So maybe mom or dad have a beer or wine or whatever, it’s there.” (Key Informant 15)

Additionally, key informants perceived both cannabis and alcohol as widely accepted among youth peer groups, and thought of them as ‘social substances’ and thus commonly used in social situations. Informants explained that peers can often influence and contribute to use, as youth tend to strive for a sense of belonging among their peers, and may feel peer pressured to use:

“Cannabis has been so normalized for kids starting at a young age, and seen as a coping tool. I think it is a real issue. I meet school kids at school who go out and smoke [with friends] because it’s better than being alone at lunch. I think for young youth, it’s more accessible than some of the other drugs, they see their peers using it, and I think they often don’t see it as problematic.” (Key Informant 11).

### Stigmatization of cannabis and alcohol use among youth

While key informants discussed their perceptions about the ways in which normalization of cannabis and alcohol use among youth increased use, they highlighted the role of stigmatization and how it simultaneously may deter youth from seeking service support. According to participants’ views, discourse perpetuates stigmatization – including stigma that specifically stems from traditional or conservative values, familial or cultural expectations, and self-stigma – and these discourses directly influence youths’ decisions to seek support for their substance use.

### Conservative values and familial expectations

Key informants suggested that traditional or conservative values among the general population regarding substance use was one of the biggest contributors to stigmatization. They explained that substance use has traditionally been stigmatized throughout history, and remains commonly associated with negative connotations, especially among youth, and conceptualized as a social or moral failing, as opposed to a public health concern:

*“*Substance use in general is probably one of the more stigmatized areas. You know, I think mental health is sort of becoming more accepted, but I still see that substance use, there’s lots of associations, negative associations and judgments, that get made around you know, sort of ‘drug user’.” (Key Informant 16).

Participants believed systemic negative social perceptions and stereotypes regarding substance use remain prevalent, and that these discourses deter youth from feeling comfortable to have open conversations about their use. One key informant further explained how they thought public discourse surrounding the illegality of substance use compounded these issues:

“I think the whole legal-illegal thing, that definitely plays a role. They recognize that this is not something that is legal for my age group, so I think they are less likely to engage in those conversations with adults, because that’s the first message that they hear, ‘well you can’t do that, that’s illegal, that’s not an okay thing for you to do for your age’.” (Key Informant 06).

Many key informants expressed their views on how family expectations placed on youth to behave a certain way, not engage in substance use, and uphold family values impacted youth’s desire to openly discuss their substance use with family members or seek support for their use. Additionally, key informants described the ways they perceived cultural values to subsequently work as barriers to seeking treatment, especially if the youth come from cultural backgrounds that particularly disapprove of substance use and may ostracize or punish them for using, as explained by one informant:

“Like here in [city name], a big conversation we’re having is that there’s a generational and cultural gap. So they’re first-born Canadians, with those kind of non-compromising values, back-home values. It’s like, how do they maneuver that in their life? And because there is still so much stigma in a lot of these cultures.” (Key Informant 10).

### Self-stigmatization

The last theme that arose related to internalized guilt and shame, and key informants suggested that they believed many youth internalized societal stigmas towards the people who use substances. For instance, one key informant suggested:

“It really comes down to being shameful and guilty. I think shame is a huge piece that’s a massive barrier. You’ve been told your whole life that drugs are bad, don’t do drugs, and then people are ostracizing you or shunning you or treating you poorly and not supporting you because of the choices that you’re making.” (Key Informant 10).

Key informants also discussed how many of the youth they work with have difficulty accepting that they have a substance use problem, and thus struggle to talk openly about it with their peers. This was illustrated by one informant who mentioned the difficulty some youth experience when coming to terms with needing support for their substance use:

“They don’t want to identify that they have a problem. I see a lot with clients, asking ‘why can my friend just have a couple drinks, and why can’t I be ok doing that?’ It’s hard for them to understand that they have addictive personalities and addictive traits, and that’s why a couple of drinks wouldn’t be enough for them because their addicted to it.” (Key Informant 04).

### Solutions identified by service providers: Challenging the discourse

A key underlying theme suggested by participants was the need to change the negative and often stigmatizing discourse around substance use. As such, key informants suggested a need to increase public awareness and knowledge, and to reform the educational system to include key messaging and education around the health effects of substance use.

### Normalizing conversations around substance use

In relation to traditional and conservative perspectives of substance use, many participants expressed the importance of changing the discourse and shifting how substance use is perceived by society and within the household. Key informants explained the need to focus on normalizing conversations around substance use so it can be spoken about more openly, and emphasized the importance of working towards reconceptualising substance use from a moral failing by shifting the discourse to look at substance use from a health perspective. Participants suggested that if there were more positive public awareness around substance use as a health issue and increased conversations around the openness of using substances, then youth would feel more comfortable and inclined to seek support:

“Taking a look at it from more of a health perspective, because I don’t think, socially, addiction or substance dependency is really viewed as a health problem… You can get around these things by identifying the treatment as a health-related need, in actuality that’s exactly what it is, and just trying to reinforce it.” (Key Informant 08).

Another informant further discussed the importance of changing the discourse by working towards normalizing substance use- related conversations:

“Traditional perspectives and viewpoints and stigma play a role, and it’s been a constant for as long as I’ve been in the field. What could help support a change in that is conversation shifting how substance use is perceived, how it’s understood, and normalizing the supports in conversation in schools, in community centres, hospitals. Just bringing that into the conversation helps normalize it, which traditionally, you wouldn’t be able to raise this topic without raising blood pressure.” (Key Informant 01).

To this end, public health educational campaigns were also discussed as important interventions, especially related to cannabis and alcohol among this demographic. Key informants suggested a need to raise public awareness thorough social media campaigns, providing youth-friendly and receptive tools, tips, and information regarding substance use, effects, and safe ways of using. The importance of providing this awareness in a way that is targeted specifically for youth was discussed. The potential effectiveness of social media platforms as a medium for increasing awareness was also expressed by one key informant:

"I think definitely public awareness through social media, that’s something that youth have been asking for. They’re like, ‘why don’t you do those Instagram or Snapchat ads? We actually read them if it’s something that’s for us’… You have to think of where they are spending their time, they are spending their time online.” (Key Informant 10).

### Reform the education system

In order to help shift the discourse to a public health lens and educate youth about the risks of substance use, participants expressed a need to reform the education system to develop awareness and normalize conversations. Many respondents emphasized the importance of making discussions about substance use and consequences of use incorporated into the curriculum, starting from an early age, and encouraging schools to generally talk more openly about substance use:

“If it’s part of the curriculum and discussed as openly as any other subject that’s important, as an important life skill to learn how to cope, I think there would be open conversations and less stigma around reaching out for help.” (Key Informant 11).

Key informants felt there is a need for a systems-level change in the education system to help inform youth on the risks associated with use, while simultaneously combatting stigma by reframing the perceptions and normalizing conversations around it. Additionally, respondents identified the need for early intervention and to be proactive by integrating substance use into the educational process early:

“The early intervention, we know that the earlier you intervene, you can address the problematic substance use before it progresses to a more serious concern, the better the outcome.” (Key Informant 06).

The need for more school-based programs where youth are offered the opportunity to openly talk about substance use, and ask questions in a non-judgmental way was highlighted by a number of participants. For instance, one key informant described the importance of speaking about substance use openly in the school system:

“I think that it should be more openly spoken about in high schools, even in universities and colleges. If they make it seem like it’s okay to have a problem, more people would be open to get the help.” (Key Informant 04).

The school system was thus described as the ideal place to provide educational supports and programs, including psycho-education, such as informational handouts, and after-school programs, including outreach from key relevant services. For instance, one key informant suggested meeting youth where they are at as a key way to reach this population:

“Regular outreach in the schools where you’re more accessible, where kids are. More education about the drugs as part of the curriculum, after school programs where they can come and talk openly without worrying about consequences.” (Key Informant 11).

Key informants further suggested the need to ensure teachers and school staff are properly trained with the appropriate information and tools to have these discussions with their students, and to be able to direct them to the appropriate services, if needed.

## Discussion

Public discourses can have a direct influence on substance initiation and continued use among youth, based on whether use of a particular substance is accepted as normative behaviour or not [21]. Understanding how certain substances become normalized, and whether and to what extent this process contributes to substance use is a necessary component for identifying effective and appropriate interventions that work towards preventing substance use from developing into more chronic use and associated issues. Our findings thus reinforce studies that suggest how substance use normalization may encourage use. For instance, social norms, increased accessibility, and positive cannabis messaging have been associated with increased cannabis use among youth [22]. Additionally, normalization has been associated with substance use experimentation, increased availability of substances, more liberal attitudes towards recreational substance use among youth and young non-users, positive portrayals of substance use in mainstream media, and relaxed policies around substance use [23]. According to the normalization framework, it is anticipated that discourse around cannabis legalization for instance may shift social norms regarding cannabis use from criminal/deviant to normative, socially acceptable behaviour, where it could lead to increased acceptability, experimentation, and use among youth [21]. Following cannabis legalization in Canada in 2018, the National Cannabis Survey (NCS) found that youth indicated that they would be more likely to try cannabis or increase their use in comparison to older adults [24]. While some studies suggest cannabis use patterns have remained relatively stable among substance use service seeking youth since legalization in Canada [25], others suggest that the normalization of cannabis given its’ legal status has subsequently increased cannabis use among youth population, even though cannabis still remains illegal for many youth [22, 26]. Specifically, in Canada, it is illegal for youth under the age of 18 in most provinces, and 21 in Quebec, to purchase and consume cannabis. Individuals can face civil penalties and/or criminal justice involvement for possessing even small amounts of cannabis, underscoring that legalization of cannabis is still associated with potential harms for youth [27]. In particular, lifetime and past-year cannabis use has increased since the beginning of federal legalization, along with the proportion of participants who thought cannabis is easy to access [22]. According to the most recent wave of the longitudinal Canadian COMPASS study of students in grade 9–12, cannabis ‘ever-use’ was significantly higher in the year after legalization, suggesting increased experimentation among youth post legalization [26]. Furthermore, in a recent national survey of Canadian youth, cannabis use had increased by nearly 6% from the year prior to cannabis legalization [28]. It is interesting to note that while the findings emphasized increased and frequent use of licit substances such as cannabis and alcohol, for many youth these substances still remain illegal. The normalization, increased accessibility and availability of such substances has made use of cannabis and alcohol seem more socially acceptable, while many youth do not understand the negative health and social consequences of use, despite its illegal status among a significant youth age group.

Our thematic results suggest that normalization of substance use without educational interventions targeting youth perceptions and knowledge about the health and social impacts of legal substances could lead youth to undermine the potential severity of cannabis and alcohol use, and subsequently lead to increased use. In terms of educational interventions, in Ontario, less than half of the high schools surveyed in the aforementioned COMPASS study indicated they were offered a drug use prevention program, demonstrating that current educational prevention programs are rare and insufficient [29]. There was a general consensus among our study participants around a perceived need for education regarding the health impacts of legalized/licit substances based on their experience providing care for youth. Interventions that target knowledge and awareness of both licit and illicit drug use harms have been shown to change knowledge and attitudes about substances among youth, however, according to two systematic reviews of school-based substance use interventions, these interventions alone do not change drug use patterns [30, 31]. Programs which combine components that aim to develop personal and interpersonal skills, as well as those which aim to reduce societal influences, have a moderate protective effect on preventing substance use [30], signifying that a combination of education-, social competence and social influence-type programs could be a potential solution.

In Ontario specifically, school-based drug education programs have evolved over the past few decades, and now offer a more multi-faceted, interactive approach, with several programs using a combination of social competency, harm reduction, and decision-making components in addition to providing key information. Yet most of these programs are not evidence-based, but rather selected based on popularity and marketing, without any consistent and mandated evaluation criteria [32]. Furthermore, Ontario schools are not obligated to provide evidence-based drug education. In primary schools across Ontario, the most widely adopted programs tend to be based on the drug abuse resistance education (DARE) model, which has been found to insignificantly impact substance use behaviours and prevalence of use among youth, particularly due to its stigmatizing philosophy of promoting resistance towards and abstinence from substance use, as opposed to promoting a harm-reduction lens and normalizing conversations around the use of substances [32, 33]. While participants in our study suggested the need for an increase in school-based educational initiatives to assist in normalizing conversations and educating youth about the harms associated with use, they also suggested the utility of social media-based campaigns as a means to provide information to help shift the discourse and reduce the stigmatizing attitudes towards use. A recent national survey of Canadians aged 16 to 30 years found that while viewing advertisements and educational messages about cannabis was uncommon (less than one-third of participants reported seeing messages about cannabis in the past year), digital media was the most common place for encountering public health messages regarding cannabis use [34]. This suggests a need for expansion of education-based prevention programs through various channels including digital and television media in addition to schools to ensure it reaches the targeted youth population. It is also imperative for these programs to involve youth in the design and implementation, as they are best-suited to identify what information should be emphasized and which platforms are most effective [35].

Our findings also suggest the influence of social acceptability around substance use at the family and peer levels are important factors, which may contribute to youth substance use. In Canadian models and frameworks of youth substance use, family is recognized as an important determinant of use [36, 37]. According to literature, adolescent alcohol and cannabis use is positively associated with perceived substance use by parents [38–42], as well as peers and close friends [39, 42]. Furthermore, relationship quality with parents (e.g., conflict), parental monitoring, and peers with anti-social behaviours have been designated as key predictors of illicit substance use initiation among youth, according to longitudinal data [43–45]. Thus, family and peers are important domains that may contribute to the normalization of youth substance use, which should be considered when developing programs.

While familial and social influences can work to perpetuate the normalization of substance use, key informants indicated that they also may simultaneously play a large role in the stigmatization that deters youth from seeking support for problematic use. Stigma acts as a persistent barrier and creates additional challenges in ensuring youth are able to access critical services when they need them, and is particularly intensified for the adolescent age groups where parental engagement and consent in substance use treatment and services is often required [12, 17, 43–46]. Therefore, it is equally important to address stigma at various levels, including at the family and peer level since adolescents are highly engaged with and exposed to these groups. Our results also emphasize the importance of targeted interventions to reduce stigma among certain groups including racial and cultural minorities, where some families hold conservative or traditional mindsets regarding substance use. In studies that identified barriers to substance use service access among refugee and migrant youth, youth were especially reluctant to use services as a result of stigma surrounding mental health and substance use due to fear of bringing shame and disappointment to their families and communities, highlighting the importance of cultural background as an important determinant of youth substance use and related outcomes [46, 47]. Evidence has documented that the use of culturally sensitive substance use treatments targeting racial minority youth are effective at reducing substance use among the target population [48]. Nonetheless, addressing stigma requires multi-level interventions targeting all levels of systemic, family, peer, internalized stigmatization, as well as the broader public discourse regarding substance use as moral failing as opposed to a public health issue. To this end, a recent federal Canadian government initiative presented a national primer aimed at reducing substance use stigma in Canada [49]; however, this document still fails to acknowledge the distinct issues faced by youth and ways that stigmatization discourages youth from accessing necessary services.

While normalization through decriminalization and liberal drug policies may reduce stigma related to drug use, stigma towards people who use drugs continue to persists [50]. The National Institute on Drug Abuse in the U.S. [51] and the Canadian Centre on Substance Use and Addiction [52] both recognize the importance of discourse in combating stigmatizing language and have therefore recommended changing the language around substance use to healthcare- and medical-oriented terminologies. Consistent with these recommendations, addiction research scholars support the use of person-first language, which reinforces an individual’s identity first and foremost, reflects the medical nature of substance use, and is recovery-oriented, which should be considered when developing programs and policies that support the reduction of stigmatization of substance use [53, 54]. This will help move away from public conceptualizations of substance use as a moral failing towards the understanding that it is in fact a public health issue, that requires services and supports.

Overall, according to our sample of direct service providers, normalization of licit substance use at the societal, family, and peer levels emerged as an important topic of concern that requires further investigation. Our findings suggest that the normalization framework is complex. For instance, normalizing discourses around youth substance use may help reduce stigma, and therefore increase youth’s desire to come forward and seek support for their use. However, if normalization is not properly balanced with education on the harms of substance use, it may have the unintended effect of increasing use. As such, service providers play an integral role and have the opportunity to not only encourage youth to have open conversations about their substance use with their peers and family, but also to change the discourse around substance use to one that is less stigmatizing by providing education to youth on the potential consequences so that they can make informed choices. Using prevention and harm reduction frameworks, service providers can foster an environment that both provides the necessary education regarding the impacts of use, while also ensuring they are supporting youth in ways they require. Evidence has suggested that policies and services that focus on prevention and harm reduction initiatives are most effective when working with youth as it provides different levels of service support that youth need, using non-judgmental, non-stigmatizing frameworks [55]. Furthermore, youth should be involved in the design of substance use educational and awareness programs [35].

According to both implementation sciences and integrated models of youth substance use care [18, 35], service providers are recognized as important stakeholders in the delivery and uptake of evidence-based interventions. Therefore, studies can gain important insight from service provider perspectives and additional knowledge regarding barriers and facilitators to service access as well as quality of care. This is especially important when designing and developing policies and programs pertinent to specific populations, such as youth.

### Limitations

Given the convenience sampling approach, our findings cannot be generalized and only reflect the perspectives of a sub-sample of youth service providers in select Ontario jurisdictions. Furthermore, the study sample’s perspectives included their own interpretation of youth and their family’s experiences. As such, responses may have been inherently biased based on service provider’s own lived and professional experiences, and this potential should be taken into consideration. In order to develop a more thorough and balanced understanding of substance use and the barriers to substance use service access among youth, future studies should aim to include a broader sample of service providers, as well as explore and compare these results with youth’s perspectives, as well as their parents/guardians/caregivers’.

## Conclusions

According to the perspectives of the service providers, our study found that the normalization of cannabis and alcohol by society, family members, and peers promoted substance use among youth, while the stigmatization perpetuated by traditional and societal perspectives and family morals deterred youth from accessing services. Both normalization of licit substance use and stigmatization therefore appear to be key components of dominant discourses around youth substance use, with important implications for the health and social impacts of substances, as well as for the formulation, development, and implementation of public health policies. At the macro level, it is important to mobilize efforts to reframe the discourse around substance use among youth to help eradicate the negative connotations and stigmatization that is associated with use, which acts as a deterrent for service support. At the micro level, appropriate and non-stigmatizing messaging and educational components must be widely adopted to ensure that accurate information regarding the health impacts of use are well known in order for youth to make informed decisions regarding their use.

## Data Availability

The datasets generated and/or analyzed during the current study are not publicly available due to privacy and confidentiality reasons of participant information.

## Abbreviation

PWLE: People with lived experience

## References

[1] Statistics Canada. Population estimates on July 1st, by age and sex. 2021. https://www150.statcan.gc.ca/t1/tbl1/en/tv.action?pid=1710000501&pickMembers%5B0%5D=1.7&pickMembers%5B1%5D=2.1&cubeTimeFrame.startYear=2016&cubeTimeFrame.endYear=2020&referencePeriods=20160101%2C20200101. Accessed 17 June 2021.

[2] Nigatu YT E-MT, Adlaf EM, Ialomiteanu AR, Mann RE, Hamilton HA. CAMH Monitor eReport 2019: Substance use, mental health and well-being among Ontario adults. 2020. https://www.camh.ca/-/media/files/pdfs---camh-monitor/camh-monitor-2019-ereport-pdf.pdf.

[3] Boak A, Elton-Marshall, T, Mann, RE, & Hamilton, HA. Drug use among Ontario students, 1977–2019: Detailed findings from the Ontario Student Drug Use and Health Survey (OSDUHS). 2020. https://www.camh.ca/-/media/files/pdf---osduhs/drugusereport_2019osduhs-pdf.

[4] World Health Organization (WHO). Orientation Programme on Adolescent Health for Health-care Providers (Module B-5). n.d. https://www.who.int/maternal_child_adolescent/documents/pdfs/9241591269_op_handout.pdf. Accessed 17 June 2021.

[5] Fischer B, Russell C, Sabioni P, van den Brink W, Le Foll B, Hall W (2017). Lower-risk cannabis use guidelines: a comprehensive update of evidence and recommendations. Am J Public Health.

[6] Schulte MT, Hser YI (2014). Substance use and associated health conditions throughout the lifespan. Public Health Rev.

[7] Winters KC, Arria A (2011). Adolescent brain development and drugs. Prev Res.

[8] Gray KM, Squeglia LM (2018). Research Review: What have we learned about adolescent substance use?. J Child Psychol Psychiatry.

[9] Whitesell M, Bachand A, Peel J, Brown M (2013). Familial, social, and individual factors contributing to risk for adolescent substance use. J Addict.

[10] Costello EJ, He JP, Sampson NA, Kessler RC, Merikangas KR (2014). Services for adolescents with psychiatric disorders: 12-month data from the national comorbidity survey-adolescent. Psychiatr Serv.

[11] Henderson JL, Cheung A, Cleverley K, Chaim G, Moretti ME, de Oliveira C (2017). Integrated collaborative care teams to enhance service delivery to youth with mental health and substance use challenges: protocol for a pragmatic randomised controlled trial. BMJ Open.

[12] Russell C, Neufeld M, Sabioni P, Varatharajan T, Ali F, Miles S (2019). Assessing service and treatment needs and barriers of youth who use illicit and non-medical prescription drugs in Northern Ontario, Canada. PLoS One.

[13] Canadian Mental Health Association (CMHA). Finding and Navigating Addiction Services for Children and Youth. 2021. https://ontario.cmha.ca/documents/finding-and-navigating-addiction-services-for-children-and-youth/. Accessed 17 June 2021.

[14] Chaim G, Henderson J. Youth System Services Review Phase 2 Report: A review of the continuum of Ontario services addressing substance use available to youth age 12–24. 2014. https://www.eenet.ca/sites/default/files/pdfs/YSSR_Phase2_Report_July28.pdf. Accessed 17 June 2021.

[15] Chaim G, Henderson JL, Brownlie EB. Youth services system review. 2013. https://www.eenet.ca/sites/default/files/pdfs/Youth-Services-System-Review-Report-Part-1-FINAL3.pdf. Accessed 17 June 2021.

[16] Brownlie EB, Chaim G, Heffernan O, Herzog T, Henderson J (2017). Youth services system review: Moving from knowledge gathering to implementation through collaboration, youth engagement, and exploring local community needs. Can J Commun Ment Health.

[17] Wisdom JP, Cavaleri M, Gogel L, Nacht M (2011). Barriers and facilitators to adolescent drug treatment: Youth, family, and staff reports. Addict Res Theory.

[18] Bauer MS, Kirchner J (2020). Implementation science: What is it and why should I care?. Psychiatry Res.

[19] Acevedo A, Harvey N, Kamanu M, Tendulkar S, Fleary S (2020). Barriers, facilitators, and disparities in retention for adolescents in treatment for substance use disorders: a qualitative study with treatment providers. Subst Abuse Treat Prev Policy.

[20] Harris PA, Taylor R, Minor BL, Elliott V, Fernandez M, O’Neal L, McLeod L, Delacqua G, Delacqua F, Kirby J, Duda SN, REDCap Consortium (2019). The REDCap consortium: Building an international community of software partners. J Biomed Inform.

[21] Sznitman SR, Taubman DS (2016). Drug use normalization: a systematic and critical mixed-methods review. J Stud Alcohol Drugs.

[22] Zuckermann AME, Battista K, de Groh M, Jiang Y, Leatherdale ST (2019). Prelegalisation patterns and trends of cannabis use among Canadian youth: results from the COMPASS prospective cohort study. BMJ Open.

[23] Parker H, Williams L, Aldridge J (2002). The normalization of ‘sensible’ recreational drug use: further evidence from the north west england longitudinal study. Sociology.

[24] Sandhu HS, Anderson LN, Busse JW (2019). Characteristics of Canadians likely to try or increase cannabis use following legalization for nonmedical purposes: a cross-sectional study. CMAJ Open.

[25] Hawke LD, Henderson J (2021). Legalization of cannabis use in Canada: Impacts on the cannabis use profiles of youth seeking services for substance use. J Subst Abuse Treat.

[26] Zuckermann AME, Battista KV, Bélanger RE, Haddad S, Butler A, Costello MJ (2021). Trends in youth cannabis use across cannabis legalization: Data from the COMPASS prospective cohort study. Prev Med Rep.

[27] Haines-Saah RJ, Fischer B (2021). Youth cannabis use and legalization in canada - reconsidering the fears, myths and facts three years in. J Can Acad Child Adolesc Psychiatry.

[28] Wiens K, Bhattarai A, Pedram P, Dores A, Williams J, Bulloch A (2020). A growing need for youth mental health services in Canada: examining trends in youth mental health from 2011 to 2018. Epidemiol Psychiatr Sci.

[29] Zuckermann AME, Gohari MR, de Groh M, Jiang Y, Leatherdale ST (2020). The role of school characteristics in pre-legalization cannabis use change among Canadian youth: implications for policy and harm reduction. Health Educ Res.

[30] Faggiano F, Minozzi S, Versino E, Buscemi D (2014). Universal school-based prevention for illicit drug use. Cochrane Database Syst Rev.

[31] Stockings E, Hall WD, Lynskey M, Morley KI, Reavley N, Strang J (2016). Prevention, early intervention, harm reduction, and treatment of substance use in young people. Lancet Psychiatry.

[32] Bruno T, Csiernik R (2020). An Examination of Universal Drug Education Programming in Ontario, Canada’s Elementary School System. Int J Ment Heal Addict.

[33] Cohen IM, Plecas D. A review of the research on the drug abuse resistance education (D.A.R.E.) program. 2005. https://www.ufv.ca/media/assets/criminology/A-Review-of-the-Research-on-the-Drug-Abuse-Resistance-Education-(D.A.R.E.pdf. Accessed 17 June 2021.

[34] Leos-Toro CFG, Meyer SB, Hammond D (2020). Cannabis health knowledge and risk perceptions among Canadian youth and young adults. Harm Reduct J.

[35] Hawke LD, Mehra K, Settipani C, Relihan J, Darnay K, Chaim G (2019). What makes mental health and substance use services youth friendly? A scoping review of literature. BMC Health Serv Res.

[36] Canadian Centre on Substance Use and Addiction (CCSA). Building on our strengths: Canadian standards for school-based youth substance abuse prevention. 2010. https://www.drugsandalcohol.ie/19992/1/Canada_Building_on_our_strengths.pdf. Accessed 17 June 2021.

[37] Public Health Agency of Canada (PHAC).The chief public health officer's report on the state of public health in Canada 2018: Preventing problematic substance use in youth. 2018. https://www.canada.ca/content/dam/phac-aspc/documents/corporate/publications/chief-public-health-officer-reports-state-public-health-canada/2018-preventing-problematic-substance-use-youth/2018-preventing-problematic-substance-use-youth.pdf. Accessed 17 June 2021. 2018.

[38] Branstetter SA, Low S, Furman W (2011). The influence of parents and friends on adolescent substance use: a multidimensional approach. J Subst Use.

[39] Chabrol H, Mabila JD, Chauchard E, Mantoulan R, Rousseau A (2008). Contributions of parental and social influences to cannabis use in a non-clinical sample of adolescents. Encephale.

[40] Miller SM, Siegel JT, Hohman Z, Crano WD (2013). Factors mediating the association of the recency of parent's marijuana use and their adolescent children's subsequent initiation. Psychol Addict Behav.

[41] Pilin MA, Robinson JM, Dow-Fleisner S, Sanchez TA, Krank MD (2021). Automatic cognitions as mediators of parental influence on adolescent cannabis use. Addict Behav.

[42] Schuler MS, Tucker JS, Pedersen ER, D'Amico EJ (2019). Relative influence of perceived peer and family substance use on adolescent alcohol, cigarette, and marijuana use across middle and high school. Addict Behav.

[43] Guo J, Hill KG, Hawkins JD, Catalano RF, Abbott RD (2002). A developmental analysis of sociodemographic, family, and peer effects on adolescent illicit drug initiation. J Am Acad Child Adolesc Psychiatry.

[44] Rusby JC, Light JM, Crowley R, Westling E (2018). Influence of parent-youth relationship, parental monitoring, and parent substance use on adolescent substance use onset. J Fam Psychol.

[45] Van Ryzin MJ, Fosco GM, Dishion TJ (2012). Family and peer predictors of substance use from early adolescence to early adulthood: an 11-year prospective analysis. Addict Behav.

[46] Gunn A, Guarino H (2016). "Not human, dead already": Perceptions and experiences of drug-related stigma among opioid-using young adults from the former Soviet Union living in the US. Int J Drug Policy.

[47] McCann TV, Mugavin J, Renzaho A, Lubman DI (2016). Sub-Saharan African migrant youths' help-seeking barriers and facilitators for mental health and substance use problems: a qualitative study. BMC Psychiatry.

[48] Steinka-Fry KT, Tanner-Smith EE, Dakof GA, Henderson C (2017). Culturally sensitive substance use treatment for racial/ethnic minority youth: A meta-analytic review. J Subst Abuse Treat.

[49] Public Health Agency of Canada (PHAC). A primer to reduce substance use stigma in the Canadian Health System. 2020. https://www.canada.ca/content/dam/phac-aspc/documents/services/publications/healthy-living/primer-reduce-substance-use-stigma-health-system/stigma-primer-eng.pdf. Accessed 17 June 2021.

[50] Skliamis K, Benschop A, Korf DJ. Cannabis users and stigma: A comparison of users from European countries with different cannabis policies. Eu J Criminol. 2020;1477370820983560.

[51] National Institute on Drug Abuse (NIDA). Words Matter - Terms to Use and Avoid When Talking About Addiction. 2021. https://www.drugabuse.gov/nidamed-medical-health-professionals/health-professions-education/words-matter-terms-to-use-avoid-when-talking-about-addiction. Accessed 17 June 2021.

[52] Canadian Centre on Substance Use and Addiction (CCSA). Changing the language of addiction [fact sheet]. 2017. https://www.ccsa.ca/changing-language-addiction-fact-sheet. Accessed 17 June 2021.

[53] Ashford RD, Brown AM, Curtis B (2018). Substance use, recovery, and linguistics: The impact of word choice on explicit and implicit bias. Drug Alcohol Depend.

[54] Broyles LM, Binswanger IA, Jenkins JA, Finnell DS, Faseru B, Cavaiola A (2014). Confronting inadvertent stigma and pejorative language in addiction scholarship: a recognition and response. Subst Abus.

[55] Logan DE, Marlatt GA (2010). Harm reduction therapy: a practice-friendly review of research. J Clin Psychol.

